# Evidence of Top‐Down Sensory Prediction in Neonates Within 2 Days of Birth

**DOI:** 10.1111/desc.70114

**Published:** 2025-12-30

**Authors:** Naiqi G. Xiao, Claire E. Robertson, Lauren L. Emberson

**Affiliations:** ^1^ Department of Psychology, Neuroscience & Behaviour McMaster University Hamilton Ontario Canada; ^2^ Department of Psychology Princeton University Princeton New Jersey USA; ^3^ Department of Psychology Colby College Waterville Maine USA; ^4^ Department of Psychology the University of British Columbia Vancouver British Columbia Canada

## Abstract

Recent studies have demonstrated top‐down modulation in perceptual cortices in infants as young as 6 months. However, it is unclear when and how this ability emerges given conflicting evidence available. This study investigates top‐down perceptual modulation by focusing on a neural signature referred to as top‐down sensory prediction, where the prediction of upcoming sensory information is exhibited in the modulation of activity in perceptual cortices. We extended a paradigm previously used to identify top‐down sensory prediction in 6‐month‐old infants to neonates. Using functional near‐infrared spectroscopy (fNIRS), we monitored occipital lobe activity in sleeping neonates held by their caregivers. The study consisted of a Learning session, where neonates were exposed to a novel auditory‐visual stimulus combination (A+V+), followed by sessions presenting occasional visual stimulus omissions (A+V−). Results showed that fNIRS channels over the occipital lobe, which were active during the Learning session, also responded to the unexpected visual omissions, indicating neonatal brains’ capability for top‐down sensory prediction. Experiment 2 confirmed that this response depended on learning the audiovisual association, ruling out non‐specific mechanisms such as heightened arousal or an increase in the visual response when a non‐specific auditory stimulus is presented. These findings offer the first evidence of top‐down modulation of visual responses in neonates, suggesting this capacity exists at birth, significantly earlier than previously thought. This study suggests that top‐down predictive processing is crucial for early perceptual and cognitive development.

## Summary

1. No prior study has explored whether the full‐term neonatal brain can modulate the visual system based on predicted sensory information or top‐down sensory prediction.
2. We utilized functional near‐infrared spectroscopy to capture activity in the occipital lobe (visual system) during unexpected omissions of visual information following an auditory cue.
3. Our findings indicate that even within the first 2 days post‐birth, the visual system of full‐term neonates reacts to unexpected visual omissions.
4. This is the earliest age at which top‐down processing or top‐down sensory prediction have been established, suggesting that this ability is foundational to development.

## 1 Introduction

Recent research in psychology and neuroscience underscores the significant role of top‐down and predictive processes in shaping perception from infancy to maturity. These top‐down processes involve the influence of higher‐level cognitive functions and representations on processing sensory input and are manifested by contextualized perceptual changes with information communicated via feedback connections (e.g., Emberson 2019; Gilbert and Li 2013). Despite the prolonged maturation of the feedback neural connections and the white matter facilitating long‐range neural connections (Cao et al. 2017; Dubois et al. 2014), recent empirical evidence, including neuroimaging and behavioral studies, indicates the emergence of top‐down processes in infancy. Studies have documented this emergence at various ages, such as 6–8 months (Emberson et al. 2015; Flaten et al. 2022; Xiao and Emberson 2023), 10–12 months (Kouider et al. 2015; Xiao and Emberson 2019). Although these findings confirm the presence of top‐down processing from around 6 months of age, it remains unclear whether this reflects an early developmental onset or if these top‐down capabilities exist even earlier. To this end, the current study investigates the capacity for top‐down processing in neonates, born full‐term, less than 2 days post‐birth using neuroimaging techniques.

Top‐down processes are essential components in mature perceptual systems by modulating, stabilizing, and optimizing perception (de Lange et al. 2018; Lamme 2018). These top‐down processes are enabled by the existence of a large amount of feedback neural connections in perceptual systems, which pass neural signals from higher‐level regions (e.g., the prefrontal cortices) or from outside the specific perceptual system (e.g., auditory to visual systems or from the amygdala) to modulate perceptual processing as well as from higher‐level or lower‐level regions within perceptual systems (Gilbert and Li 2013; Friston 2005; Lamme et al. 1998; Rao and Ballard 1999). We consider the changes in perceptual processing (both behaviorally and neurally) arising from information traveling using feedback connections to be top‐down perceptual modulation (Emberson 2019; Gilbert and Li 2013). Although our understanding of these top‐down or feedback mechanisms in the mature brain is still emerging, research has established their role in supporting diverse perceptual functions (Gandolfo and Downing 2019; Kok et al. 2017; Oliva and Torralba 2007; Squire et al. 2013; Summerfield et al. 2006). The significance of feedback connections is highlighted by anatomical findings indicating a predominance of feedback over feedforward connections in regions traditionally associated with bottom‐up processing. For instance, the primary visual cortex (V1) receives feedback from a multitude of higher visual areas (e.g., V2, V4, MT, etc.) and is also involved in feedback loops with subcortical structures like the LGN. These feedback connections highlight the substantial influence of top‐down modulation even in areas primarily engaged in basic sensory processing (Gilbert and Li 2013).

In addition, recent work has been investigating whether top‐down processing can influence perception earlier in development. Since top‐down processes require feedback neural connections and neural connectivity, particularly long‐range neural connectivity, these systems undergo a prolonged postnatal development (Cao et al. 2017; Dubois et al. 2014). As a result, it had been thought that top‐down processes might not be available to modulate perception in infancy. Theoretical accounts have suggested different timelines for their emergence, ranging from later in infancy (Amso and Scherif 2015) to childhood (Christiansen and Chater 2016). Expanding from these views about the limitation of top‐down processing early in development, accumulating behavioral and neuroimaging evidence suggests the presence of top‐down modulation in infancy. A pivotal study by Emberson et al. (2015) demonstrated that infants exposed to novel audiovisual pairs and subsequently presented with either familiar pairs or auditory stimuli alone showed activity in early and mid‐level visual regions in response to the audio cue alone. This finding indicates that the infant visual system is subject to top‐down influences via feedback connections, a notion supported by similar EEG studies (e.g., Kouider et al. 2015). Parallel findings in language development research (Bortfeld et al. 2005) reinforce the concept of early top‐down or feedback processing. Subsequent studies have investigated the functionality of these signals using imaging and behavioral methods (e.g., Emberson et al. 2017; Jaffe‐Dax et al. 2020; Xiao and Emberson 2023), their potential variations in populations with a higher risk of developmental challenges (e.g., Emberson et al. 2017), and their relationship with perceptual development (e.g., Xiao et al. 2024). This growing body of literature not only confirms the presence of top‐down processes early in development but also highlights the necessity of considering these processes in understanding visual and language development.

A critical yet unexplored question pertains to the developmental origins of top‐down processing abilities. Considering the extended maturation period of long‐range neural connections in the brain, a key inquiry emerges: Does top‐down processing emerge developmentally within the first 6 months of life, or is it a capacity of the brain from its earliest developmental stages? Investigating top‐down sensory prediction capacities at birth is essential to addressing this question.

Previous research with human neonates has revealed that neonates are capable of learning, retaining various forms of memories (e.g., habituation, associative learning, & long‐term memory), and engaging higher‐level neural regions during cognitive tasks. There are findings where neonates have retained memories from their gestational period as fetuses (e.g., Gonzalez‐Gonzalez et al. 2006). Additionally, there are numerous studies that neonates engage in learning of simple associations even while asleep (for a review, see Tarullo et al. 2011). There is also significant research on habituation in preterm infants, indicating their capacity to habituate to new stimuli, although premature birth and associated risk factors can affect this ability (Dumont et al. 2017; Kavsek and Bornstein 2010). This body of work, encompassing studies on fetuses, preterm infants, and full‐term neonates, highlights their substantial learning and memory capabilities. These cognitive skills may be foundational for neonates to engage in top‐down processes that modulate perceptual processing. However, the extent to which top‐down processes are effective at birth remains largely unexplored.

Evidence suggests that top‐down processes in perception develop during infancy, raising questions about their availability earlier in development. For instance, Xiao and Emberson (2019) found that only 9‐month‐olds, not 6‐month‐olds, could enhance face perception using associated emotional vocal sounds, indicating a developmental progression in top‐down processing. Similarly, Nakashima et al. (2021) observed that backward masking, a phenomenon reliant on recursive neural connections within visual cortices, did not manifest until 7 months, suggesting developmental changes in these perceptual capacities. Similarly, there are theoretical proposals that top‐down processes and/or prediction that will influence perceptual processing emerges later in development (Amso and Scherif 2015; Christiansen and Chater 2016). Thus, there are both empirical findings and theoretical proposals that top‐down sensory prediction may emerge and exhibit developmental changes in infancy and beyond.

Although emerging evidence suggests that neonates, including those born prematurely, engage in predictive processing, the question of whether this involves top‐down mechanisms remains open. Several recent studies have explored the neural correlates of predictive processing in neonates, but their designs limit the interpretability of their findings regarding top‐down control. For example, Dumont et al. (2022) used Diffuse Correlation Spectroscopy to measure blood flow changes in the somatosensory cortex of premature neonates during a vibrotactile stimulation‐omission paradigm. They found that, after a learning phase, the neural response to the omission of the vibrotactile stimulus closely resembled the response to the stimulus itself, suggesting a form of predictive processing. However, because this study employed a unimodal design involving only vibrotactile stimuli, it is difficult to determine whether this prediction involved top‐down processing, such as feedback connections from higher‐order cortical areas, or a more basic, potentially reflexive, mechanism.

Similarly, Dall'Orso et al. (2021) used fMRI to examine neural responses in full‐term neonates to auditory stimuli that predicted a vibrotactile stimulus producing movement. They found similar neural signatures in the somatosensory cortex for both the vibrotactile stimulus itself and the predictive auditory cue. This again points to sensory prediction. However, given the established cortical hierarchy in the auditory system, where auditory information is processed in the temporal lobe before being relayed to the frontal and somatosensory cortices (Kaas and Hackett 2000; Rauschecker and Scott 2009), these findings likely reflect feedforward, rather than top‐down, neural connections. Although these studies provided compelling evidence for predictive processing in neonates across both unimodal and cross‐modal paradigms, definitive evidence for top‐down perceptual processing involving feedback connections and higher‐order cortical areas remains elusive.

This ambiguity regarding top‐down processing in neonates motivated the current study. Although previous research indicates the emergence of prediction for specific stimuli during the first year (e.g., Flaten et al. 2022; Xiao and Emberson 2019, 2023; Xiao et al. 2024; Zhang et al. 2018), studies in neonates have primarily focused on somatosensory responses, limiting inferences about top‐down modulation, which requires feedback connections within a cortical hierarchy. To address this gap, we conducted two experiments to investigate responses to an auditory cued visual omission in the visual cortex of neonates using functional near‐infrared spectroscopy (fNIRS). Experiment 1 examined whether an auditory cue associated with a visual stimulus could activate the visual cortex during an unexpected visual omission. We hypothesized that if neonates engage in top‐down sensory prediction, the visual cortex would respond to the auditory cue in the absence of the visual stimulus, similar to patterns observed in older infants and adults (e.g., Emberson et al. 2015; den Ouden et al. 2009). In contrast, if top‐down sensory prediction is absent, the visual cortex would respond only to the presence or absence of the visual stimulus itself. Experiment 2 addressed the possibility that the neonatal occipital cortex might respond to auditory stimulation regardless of whether it predicts a visual event or not. The findings of this study will provide crucial insights into the developmental origins of top‐down processing.

## 2 Experiment 1

### 2.1 Participants

Sixteen neonates participated in the current Experiment (10 females and six males, no further demographic information was collected). These participants were all full‐term (>37 gestational weeks) healthy newborns. Their age ranged from 11 to 56 h after birth with a mean age of 27.61 h. Five additional neonates participated in the current study but were excluded from data analyses due to the failure to record neural signals (*n* = 2) or failure to complete the study due to fussiness (*n* = 3). Though participants were of diverse racial backgrounds, including Black, Eastern Asian, Southeastern Asian, Latino, and White, participants’ ethnicity was not explicitly disclosed by their caregivers. The head circumference of these participants ranged from 32 to 36 cm. Participants were recruited from the maternal unit at a local hospital in a North American city.

The sample size was determined via a power analysis based on data from a pilot study (*N* = 6) using the identical experimental design. The power analysis focused on a two‐tailed one‐sample *t*‐test evaluating changes in oxygenated hemoglobin concentration in occipital channels (over visual cortices) in response to the auditory cue alone (i.e., the visual absent condition). With an observed effect size of *d* = 0.96 from the pilot study, an alpha level of 0.05, and a desired power of 0.80, the analysis yielded a required sample size of 10.61. The current sample size (*N* = 16) exceeds this estimate, providing sufficient power to detect the effect of the predictive auditory stimulus on visual cortex activity. This analysis was conducted using the “pwr” package in R. The six infants from the pilot study whose data were used for the power analysis were not included in the final sample reported and analyzed in the main study.

### 2.2 Procedure and Materials

All neonates participated in the study while asleep. The neonates were held by their caregivers (five mothers, two grandmothers, and nine fathers) while participating in the current study. The study session comprised two consecutive sessions: an audio‐visual learning session and a test session. In the learning session, participants heard a cis‐gender woman voicing /ba/ (800 ms duration), which was followed by a white light stimulation (800 ms duration). The audio and visual stimulation overlapped for 400 ms, resulting in a 1.2‐s duration of the audio‐visual event. Within each learning block, the audio‐visual event was repeated 18 times with inter‐event intervals, whose duration varied between 1 and 1.5 s. Participants experienced the same audio‐visual association for six blocks in the learning session. Each block was followed by a silent period, where no audio or visual stimulation was presented. The silent period lasted for a variable duration between 20 and 30 s. On average, the duration of the learning session was 6.79 min.

**FIGURE 1:**
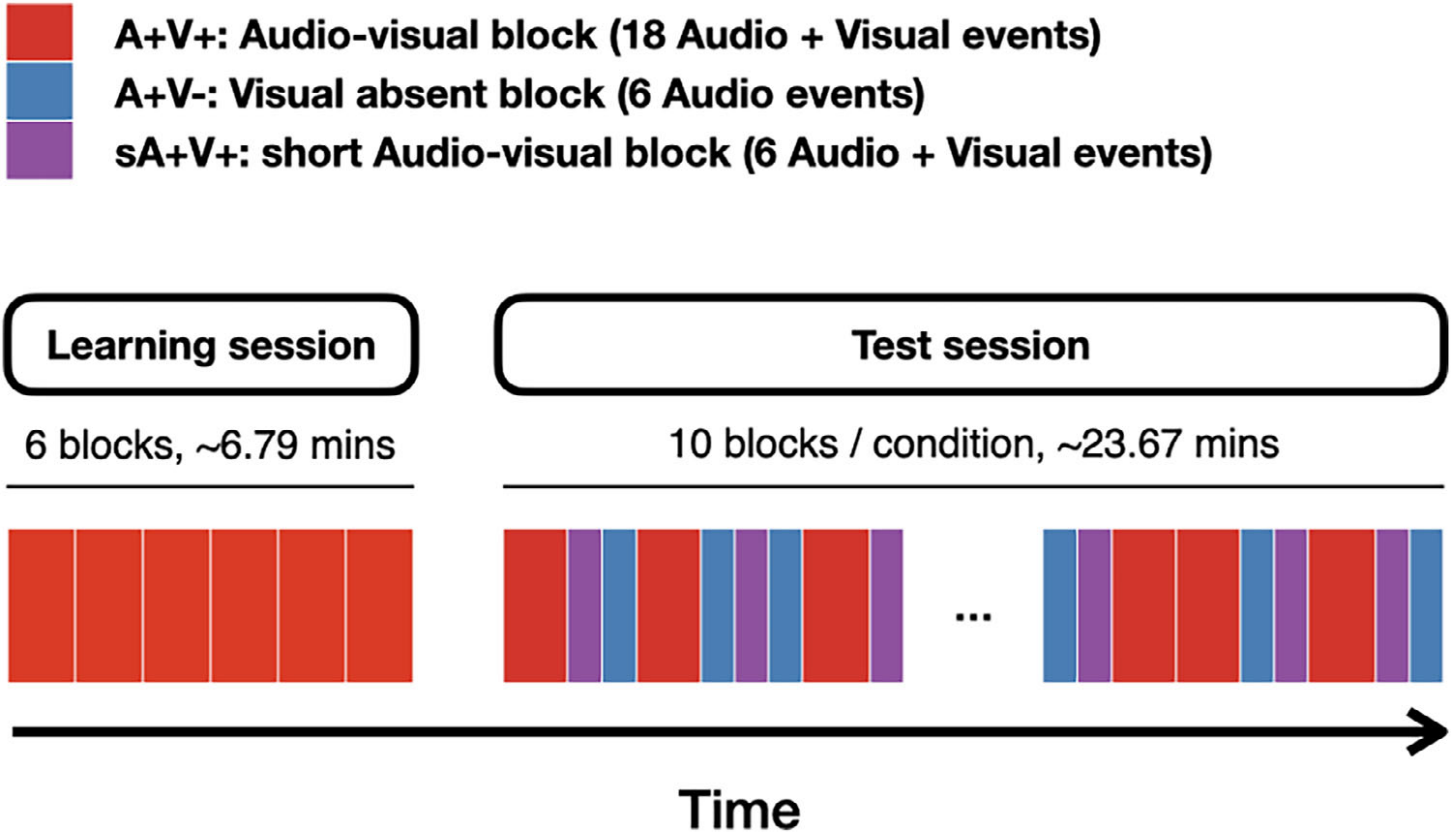
Schematic presentation of the experimental procedure of Experiment 1.

The test session followed the learning session. In the test session, participants experienced three types of experimental blocks: ***Audio‐visual block***, ***short Audio‐visual block***, and ***visual absent block***. The ***Audio‐visual block (A+V+)*** was identical to the audio‐visual learning block, thereby strengthening participants’ learning of the association between sound and light stimulation. The ***Visual absent block (A+V***−**)** was the critical condition to examine top‐down modulation in the neonatal brain. In this block, we only present the same audio stimulus without visual stimulation. Participants would hear the same sound (/ba/) played six times with an inter‐event interval of random duration between 1.4 and 1.9 s (i.e., the same pace as stimulation in the learning blocks, longer inter‐event interval is due to the absence of the light). Lastly, the ***short Audio‐visual block (sA+V+)*** was identical to the ***Audio‐visual block (A+V+)***, where both audio and visual stimuli were presented in tandem with the exception that the audio‐visual event was played only six times. This matched the stimulation in the ***Visual absent block (A+V***−**)** and served as a reference condition to gauge the impact of top‐down modulation expected in the Visual absent block. These three types of blocks were presented in random order for 10 iterations. The A+V+ block lasted for about 40s, and the A+V− and sA+V+ blocks lasted for ∼13.45s. Like the procedure in the learning session, each block presentation was followed by a silent period, whose duration varied between 20 and 30 s. The total test session lasted for about 23.67 min (Figure 1).

### 2.3 FNIRS Recording and Channel Placement

**FIGURE 2:**
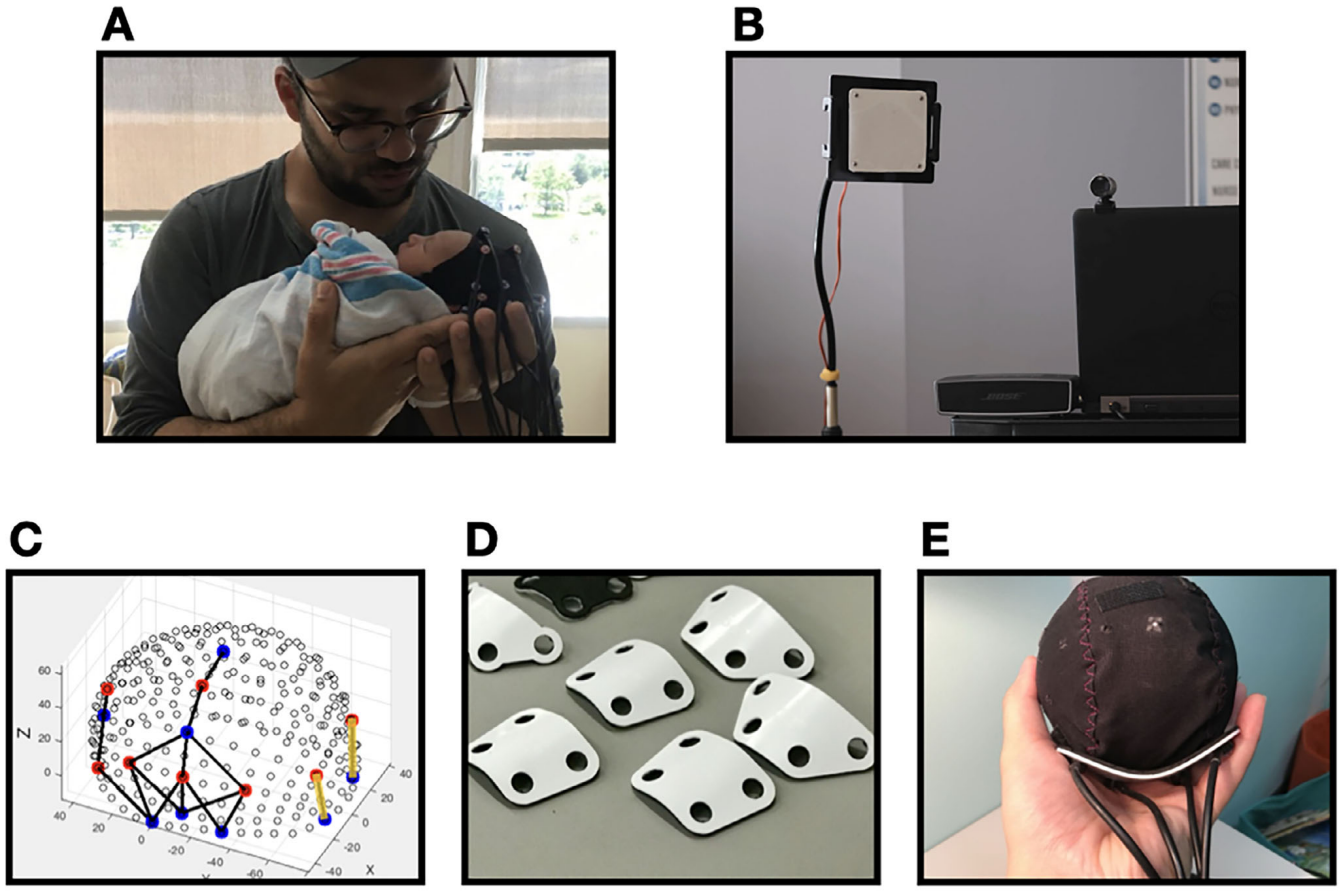
A demonstration of how neonates participated in the study while being held by their caregiver (Panel A). Experiment stimulation apparatus (LED matrix and speaker, Panel B). The placement of sources (red dots), detectors (blue dots), and recording channels (black lines) on the recording cap. The two highlighted channels are the ones representing the occipital channel (Panel C). We constructed plastic sensor holders to stabilize the placement of occipital sensors and to support participants' heads while they were being held. These holders were tailored to fit sensor locations on caps of various sizes. We designed each holder specifically for a cap of a particular size, ensuring that every participant used a holder that matched their cap size (Panels D & E).

We used the Gowerlabs NTS fNIRS system with LED light source at 780 and 850 nm to record the optical signals correlated with neonates’ brain activity. The recording frequency was 10 Hz. We used recording caps from Easycap (Wörthsee, Germany) to hold fNIRS sensors. The cap sizes ranged from 31 to 38 cm circumference (Gowerlab_Preterm_12‐16). This range allowed us to select the cap that best suited the size of the participants’ heads. The fNIRS sensors include eight sources and eight detectors, which constructed recording channels covering the occipital (two channels), left temporal (four channels), frontal (three channels), left parietal lobes (two channels) as well as the regions between these regions (six channels) as created by Gowerlabs using age and head‐size appropriate atlases (see Panel C, Figure 2). The placement of the sensors was determined according to the international 10–20 system. Specifically for the two occipital channels, the focus of the current study, were formed vertically between PO3 and OI1 and one between PO4 and OI2. The distance between the source and detector of a channel was 3 cm and did not vary with cap size. Having constant distance between the source and detector guarantees the comparability of hemodynamic recordings across individuals.

Because the current study aimed to explore how neonates’ visual response can be activated by learned audio cues, brain activity in visual cortices, which are primarily in the occipital lobe, was the focus of interests in the current study. Given that goal and also the relatively small size of neonates’ heads relative to the channel size (3 cm), our primary goal in channel placement was that the occipital channels could cover participants’ occipital lobe. We carefully placed them slightly above the inion. During the study, because most participants had their caregivers holding them at the back of their heads, their caregivers’ hands likely touched the recording sensors which had a tendency to cause artifacts in the recorded data. To effectively reduce this kind of interference, we specifically built a plastic holder to secure the sensors and to improve participants’ comfort (Figure 2).

Due to the variability in newborn head shape and the fixed geometry of the fNIRS caps, precise co‐registration of channels over non‐occipital brain regions (e.g., frontal, temporal, and parietal lobes) was challenging. Although occipital channels were secured reliably and centered in the cap placement (i.e., we prioritized placing the occipital channels over their anatomical targets first), variations in head shape, particularly dolichocephaly (elongated head shape), could shift the location of other channels relative to their intended targets. Further, the constraints of the testing environment within the maternal unit precluded the use of rigorous co‐registration procedures. Consequently, the spatial specificity of these non‐occipital channels could not be guaranteed across participants. Therefore, to ensure reliable and spatially specific neural recordings, the analyses focused primarily on the occipital channels, which were directly relevant to our research question.

After placing the recording cap, we instructed caregivers to sit conformably in a chair while holding their newborns. Then, we performed a calibration process to optimize the intensity of infrared light in each source before the study started. All neonates participated in the study while their eyes were closed. Infants were presumed to be asleep throughout the study, as consistently indicated by sustained eye closure observed during the experimental sessions.

### 2.4 Experimental Apparatus

The visual stimulation was created by an 8‐by‐8 light‐emitting diode (LED) matrix (Adafruit NeoPixel NeoMatrix), placed 30 to 40 cm above participants’ eyes. The auditory stimulation was delivered via a speaker placed 30 to 40 cm from participants’ head. Before the experiment started, we would adjust the LED brightness and sound volume according to the lighting and noise level of the testing environment for each participant. For example, we would tune down the brightness if the testing room was already dark (e.g., tested in the late afternoons). We measured the brightness and volume level based on the sensors on a smart phone (iPhone 7 plus, Apple) with a mobile phone app (Science Journal, Google). In the app, LED brightness was measured in Exposure Value (EV), and the volume was measured in decibels (dB). At the beginning of each experiment, we first measure the environment brightness level (i.e., when the LED was off), which ranged between 0 and 0.5 EV. Then, we adjust the brightness of LED by placing the smart phone camera to the LED. The LED brightness was determined when app showed brightness level around 6 EV. Similarly, the speaker volume was set between 40 and 50 dB, and environmental sounds were between 20 and 25 dB. Stimulus timing and communication with fNIRS recording (via serial port) were controlled by a Python script running on a Raspberry Pi 3 Model B computer.

We also video recorded participants throughout their study sessions. We relied on these videos to label excessive motion from the caregivers who held participants and that from participants. The motion was assessed by a researcher who did not know the study hypothesis or contents. These motion labels were later used to exclude fNIRS recordings of given trials from final analysis. Among the total 30 blocks, we excluded fNIRS recordings of one block from four participants, three blocks from one participant, and eight blocks from one participant.

### 2.5 Data Preprocessing

We used SPM for fNIRS toolbox (SPM‐fNIRS version 3, Tak et al. 2016) to perform a series of preprocessing steps, which converted optical density data into hemoglobin concentration changes for each channel by using the modified Beer–Lambert Law (Delpy et al. 1988) based on wavelength of emitted light from the source probes (780 and 850 nm). The differential pathlength factor (DPF) used in calculating the changes in the concentration of oxyhemoglobin (HbO) and deoxyhemoglobin (HbR) were 5.2781 for 780 nm and 4.28 for 850 nm wavelengths.

With the resultant hemoglobin concentration change data, we have performed the following processing in order: (1) ***motion artifact correction***. We used the movement artifact reduction algorithm in SPM‐fNIRS (MARA, Scholkmann et al. 2010) to correct motion artifacts in hemoglobin concentration change data. Motion artifact correction was corrected on a channel‐by‐channel basis with a *time window* of 1 s and a *smoothing factor* of 5. (2) ***detrending the signal*s**. We applied a default detrend algorithm (Discrete Cosine Transform, DCT) in SPM‐fNIRS to correct drift in the time series of hemoglobin changes data with the cutoff period of 128 s. (3) ***frequency filtering***. We used a Butterworth bandpass filter provided by HOMER2 (hmrBandpassFilt.m, Huppert et al. 2009) to filter out high‐frequent signals. The high pass frequency was 0 Hz, and the low pass filter frequency was 1 Hz. (4) ***baseline correction***. Oxygenated and de‐oxygenated hemoglobin concentration data for each block were baseline‐corrected using a 5‐s pre‐stimulus period recorded immediately before block onset. This 5‐s pre‐stimulus period was at the end of a longer silent baseline ensuring that the hemodynamic responses to the previous trial or block had returned to baseline responsivity. The resulting data represent the change in hemoglobin concentration relative to this baseline period, effectively removing any pre‐existing offset or drift. These baseline‐corrected hemoglobin concentration data were then analyzed using one‐sample *t*‐tests against zero to determine whether the neural responses elicited by auditory or visual stimuli increased significantly from baseline activity.

### 2.6 Results

We averaged the processed hemoglobin concentration change data of each time point across trials for each condition within each participant. The averaged data were used to conduct statistical analyses evaluating the top‐down neural modulation in neonates. Specifically, we chose a time window of 40 s after stimulation onsets of each block. We chose this time window for the following reasons: (1) this time window covered the entire stimulation period for all conditions and a subsequent period for the resulting hemodynamic response. Thereby the hemodynamic changes should index corresponding neural activity in the target brain region. It should be noted that the three types of bocks (A+V+, A+V−, and sA+V+) varied in their stimulation periods: ∼40s for the A+V+ block and ∼13.45s for the A+V− and sA+V+ blocks. We chose the same analysis window for all blocks to ensure the hemodynamic responses results were comparable across conditions, (2) the time course of hemodynamic signals from neonates’ brains is yet to be well established, and the peak response latencies of hemodynamic signals vary substantially across individuals (Gervain et al. 2011). Averaging signal across a wide analysis window would improve the likelihood to capture the neural response across participants. Similar wide time window approach has been used in previous fNIRS studies with neonates and young infants (e.g., Gervain et al. 2016; Yang et al. 2016). Finally, because previous neonate studies showed that Oxygenated hemoglobin concentration changes consistently indexed newborns neural responses (for a review, see de Roever et al. 2018), we focused our analyses on Oxygenated hemoglobin concentration changes.

To maximize the sensitivity of our analyses to visual cortex activity and minimize potential artifacts, we employed a channel selection procedure analogous to localizer methods in fMRI (functional Magnetic Resonance Imaging) research and similar to the approach described by Powell et al. (2018). Because caregivers supported the back of the neonates' heads during the experiment, some occipital channels may have been affected by hand movements or pressure. Therefore, for each neonate, the occipital channel exhibiting the largest oxygenated hemoglobin response to the audio‐visual stimulation during the Learning session was selected as the most representative of visual cortex activity. Subsequent analyses of the Test session data were then restricted to these individually selected channels. This ensured that the channel selection process, based on Learning session data, was independent from the Test session data used in the primary analyses and minimized the influence of caregiver‐related artifacts.

Furthermore, focusing on a single, optimally responsive occipital channel per neonate was justified by several factors. First, examining activity within this same channel during the test phase was essential for detecting top‐down modulation, as the hypothesized top‐down signal would manifest as an increase in visual cortex activity relative to the baseline. Second, our primary research questions focused specifically on activity within the visual cortex, making a comprehensive mapping of other brain regions unnecessary. Lastly, signal quality and neuroanatomical localization are most reliable within the occipital lobe, given the challenges of fNIRS co‐registration in other areas. This targeted approach allowed for a precise examination of relative response patterns within and between subjects in our region of interest, the occipital lobe, rather than attempting a broader, less precise cortical mapping.

**FIGURE 3:**
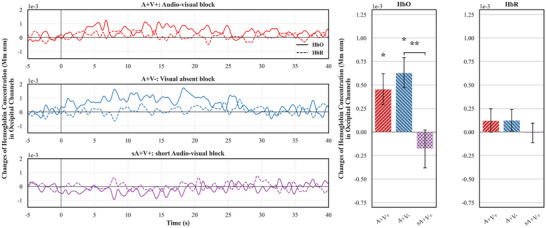
Changes in hemoglobin concentration in Occipital channels for each condition. The left panel shows the time course results for oxygenated hemoglobin concentration changes in solid lines and de‐oxygenated hemoglobin concentration changes in dashed lines. The right panel shows the averaged hemoglobin concentration changes within a 40‐s time window after the block onsets. Asterisks indicated significant hemoglobin concentration changes as compared with baseline. The asterisk between the A+V− and sA+V+ conditions indicates a significant difference in oxygenated hemoglobin concentration changes between the two conditions. Error bars represent one standard error of the mean.

Compared to the baseline, we have found a significant increase in oxygenated hemoglobin in the ***audio‐visual condition*** (A+V+, *one‐sample t*(15) = 2.81, *p* = 0.013, Cohen's *d* = 0.70, Figure 3). This finding suggests that neonates showed visual response to the light stimulation. More importantly, we also found a significant increase in oxygenated hemoglobin concentration in the ***visual absent condition*** (A+V−, *one‐sample t*(15) = 3.94, *p* = 0.001, Cohen's *d* = 0.98), where neonates only heard six audio events that predicted the visual stimulation without actual visual stimulation. The visual response to the predictive sound indicated the existence of top‐down sensory modulation at birth. The visual cortices respond to not only sensory input but also the predictive signals from a different sensory modality (i.e., auditory).

Interestingly, with reduced visual stimulation (light for six times) in the ***short audio‐visual condition*** (sA+V+), we did not find reliable visual responses in neonates (*one‐sample t*(15) = −0.89, *p* = 0.385, Cohen's *d* = 0.22), despite the presence of predictive sound and visual stimulation. The absence of visual response in this condition was likely caused by the combination of two mechanisms: (1) the adaptive nature of top‐down processes and (2) the weakened visual stimulation. The former mechanism suggests that top‐down modulation might be effectively engaged only when the sensory stimulation is unavailable. However, when visual stimulation was presented, the top‐down processes seem to cease functioning or visual cortices stop respond to top‐down signals, therefore leading to a significantly decreased oxygenated hemoglobin concentration in the ***short audio‐visual condition*** from the ***visual absent condition*** (sA+V+ vs. A+V−, *paired‐sample t*(15) = 3.17, *p* = 0.006, Cohen's *d* = 0.79). The latter mechanism suggests that the visual stimuli (six light stimulation) in the ***short audio‐visual condition*** might be too weak to stimulate measurable oxygenated hemoglobin concentration changes in neonates’ brain. In comparison with the oxygenated hemoglobin concentration in the ***audio‐visual condition*** (with 18 light stimulation), the ***short audio‐visual condition*** showed a significantly less activation (*paired‐sample t*(15) = 3.08, *p* = 0.008, Cohen's *d* = 0.77).

In line with other neonatal fNIRS studies (for a review, see de Roever et al. 2018), we found that Oxygenated hemoglobin concentration was the most sensitive measure to index brain activity. None of the effects that we found in oxygenated hemoglobin concentration was evident in de‐oxygenated hemoglobin concentration (Figure 3, *p*s > 0.290, Cohen's *d*s < 0.27), suggesting the changes in oxygenated hemoglobin concentration index neonates’ neural activity and their effects represented the availability of top‐down sensory modulations at birth.

Although the current study observed no significant decrease in deoxygenated hemoglobin concentration, a common hemodynamic correlate of neural activity in adults, this is common in infant fNIRS studies (for a review, see Issard and Gervain 2018). Moreover, the anti‐correlational relationship between deoxygenated hemoglobin and neural activity commonly observed in adults is less consistent in neonates. Watanabe et al. (2017) documented a positive covariation between oxygenated and deoxygenated hemoglobin during early brain development, suggesting an in‐phase relationship likely attributable to the complex interplay of metabolic and neurovascular processes during this period. Importantly, however, our findings revealed a clear *anti‐phase* relationship between oxygenated and deoxygenated hemoglobin, with the two signals changing in opposite directions (Figure 3). This anti‐phase pattern, despite the typical in‐phase coupling in neonates, strengthens the argument that the observed hemodynamic responses reflect neural activity rather than systemic physiological artifacts like heart rate or vascular changes.

Experiment 1 provided evidence that auditory cues alone, after being paired with visual stimuli, can elicit responses in the visual cortex. However, to confirm that this effect is truly a result of prediction arising from audiovisual associative learning, rather than other potential non‐specific explanations, a further control experiment is necessary. These potential non‐specific explanations could include general arousal elicited by any sound, or a more direct stimulus‐nonspecific activation of the visual cortex by auditory input, potentially mediated by existing anatomical connections between these regions and unrelated to learned associations (e.g., Eckert et al. 2008). Experiment 2 was therefore conducted to examine these alternatives and isolate the contribution of predictability.

## 3 Experiment 2

In the current study, we aimed to address the possibility that neonates’ brain activity is yet functionally modularized. Thereby, their Occipital cortices may respond to auditory stimulation even if the auditory stimulation does not predict an upcoming visual stimulus.

### 3.1 Participants

Sixteen neonates participated in the current Experiment (nine females and seven males). These participants were all full‐term (>37 gestational weeks) healthy newborns. Their age ranged from 9 to 55 h after birth with a mean age of 30.18 h. Similar to Experiment 1, participants were sleeping and held by their caregivers (4 mothers and 12 fathers). Three additional neonates participated in the current study but were excluded from data analyses due to the failure to record neural signals (*n* = 2) or failure to complete the study (*n* = 1). Participants were recruited from the maternal unit at a local hospital in a North American city. Though participants were of diverse racial backgrounds, including Black, Eastern Asian, Southeastern Asian, Latino, and White, participants’ ethnicity was explicitly disclosed by their caregivers. None of the participants in Experiment 2 participated in Experiment 1. The sample size for Experiment 2 was based on the same power analysis conducted for Experiment 1.

### 3.2 Procedure

In the current experiment, we designed three types of blocks: ***Audio only condition*** (uA+V−, “u” stands for unpredictive), ***Visual only condition*** (uA−V+), and ***short Audio only condition*** (suA+V−, “s” stands for short). The ***Audio only condition*** (uA+V−) and ***Visual only condition*** (uA−V+) were similar to the ***Audio‐visual condition*** (A+V+) in Experiment 1, where stimulation was presented for 18 times (∼40s). The differences were that the ***Audio only condition*** (uA+V) only presented audio stimulation (i.e., /ba/) and the ***Visual only condition*** (uA−V+) only presented visual stimulation (LED light). The ***short Audio only condition*** (suA+V−) was identical to the critical ***Visual absent condition*** (A+V−) in Experiment 1, where the sound of /ba/ was played six times without visual stimulation. The only differences between the ***short Audio only condition*** (suA+V−) and the ***Visual absent condition*** (A+V−) in Experiment 1 lay in that the predictiveness of the audio sound: the former did not predict visual stimulation, but the latter did. The number of audio and visual stimulation in Experiment 2 was determined by matching the number of stimulations in Experiment 1. The three types of blocks were presented in random order for 10 iterations.

If the visual response found in Experiment 1 was stimulated by any auditory stimulation, rather than by the sound associated with visual events, we should observe visual responses in all three types of blocks. Alternatively, because the sound in Experiment 2 did not predict anything visually, we should only observe visual response when visual stimuli were presented (i.e., in the ***Visual only condition***).

### 3.3 Results

**FIGURE 4:**
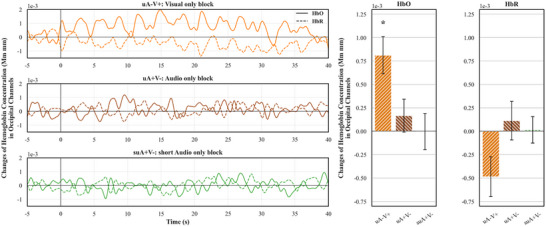
Changes in hemoglobin concentration in occipital channels for each condition. The left panel shows the time course results for oxygenated hemoglobin concentration changes in solid lines and de‐oxygenated hemoglobin concentration changes in dashed lines. The right panel shows the averaged hemoglobin concentration changes within a 40‐s time window after the block onsets. Asterisks indicated significant hemoglobin concentration changes as compared with baseline. Error bars represent one standard error of the mean.

We performed identical preprocessing to convert optical density data into hemoglobin concentration change data. And we used the same analysis pipelines to process the time‐course data. As shown in Figure 4, we found a significant increase in oxygenated hemoglobin in the ***Visual only condition*** (uA−V+, *one‐sample t*(15) = 4.08, *p* < 0.001, Cohen's *d* = 1.02), indicating a reliable visual response to light stimulation. However, we did not find any change of oxygenated hemoglobin in the ***Audio only condition*** (uA+V−, *one‐sample t*(15) = 0.95, *p* = 0.359, Cohen's *d* = 0.24) or ***short Audio only condition*** (suA+V−, *one‐sample t*(15) = −0.02, *p* = 0.987, Cohen's *d* < 0.01). Moreover, the effect that we found with oxygenated hemoglobin change did not occur in the changes in de‐oxygenated hemoglobin, indicating these oxygenated hemoglobin changes indexed neural activity. Together, these results indicated that neonates’ Occipital cortices respond specifically to visual stimulation, but not to auditory stimulation.

To further evaluate the effect of top‐down signals in modulating visual responses, we compared the oxygenated hemoglobin changes in the ***Visual absent condition*** (A+V− in Experiment 1) with that in the ***short Audio only condition*** (*su*A+V− in Experiment 2). It should be noted that the stimulation presented in these two conditions were identical (six audio sounds). The only difference lies in the predictiveness of the audio sound. Specifically, the sound in the ***Visual absent condition*** (A+V− in Experiment 1) predicted visual stimulation, whereas the sound in the ***short Audio only condition*** (suA+V− in Experiment 2) did not. The comparison between the two would serve as the best evidence for top‐down sensory modulation at birth. Thus, we performed an *independent‐sample t‐*test. The results showed a significantly larger amount of oxygenated hemoglobin concentration in the ***Visual absent condition*** (A+V− in Experiment 1) than in the ***short Audio only condition*** (suA+V− in Experiment 2, *t*(30) = 2.53, *p* = 0.017, Cohen's *d* = 0.89). To confirm this finding was not an artifact of the 40‐s analysis window, we repeated this analysis using a shorter 20‐s window, which also yielded a significant difference (*t*(30) = 2.46, *p* = 0.020, Cohen's *d* = 0.87). This contrast demonstrates that the observed auditory‐evoked visual response is contingent upon learning the audiovisual association, rather than reflecting a non‐specific mechanism such as heightened arousal.

## 4 General Discussion

Although top‐down sensory prediction has been observed in infants from 6 months (e.g., Emberson et al. 2017, 2015), its developmental origins are unclear. This study investigated this capacity in neonates using a modified version of a well‐established audiovisual paradigm. In Experiment 1, after neonates learned to associate a sound with a subsequent visual stimulus, fNIRS channels over the occipital lobe, functionally identified as responsive to visual input, showed significant activation even when the visual stimulus was unexpectedly omitted during a testing phase. Such responses to omission are indicative of top‐down sensory prediction. Importantly, Experiment 2 demonstrated that a separate group of neonates, who did not undergo this associative learning, exhibited no such visual activation in response to the sound alone. This contrast confirms that the effect in Experiment 1 was due to learned predictive processing, not a non‐specific auditory response or arousal. These findings provide the first evidence for top‐down modulation of visual responses in neonates, indicating this capacity is present at birth, substantially earlier than previously shown. This is especially noteworthy since the neonatal visual system is relatively immature compared to other sensory modalities like audition (Atkinson 2002; Graven and Browne 2008), and it has significant implications for understanding early sensory and cognitive development.

The current finding substantially expands past work examining prediction and responses to unexpected sensory omissions in neonates. Prior work has shown evidence of sensory prediction, for instance, using tactile stimuli with recordings in somatosensory and motor cortices (e.g., Dall'Orso et al. 2021; Dumont et al. 2022). As reviewed in the Introduction, while these studies confirmed neonates’ capability to generate sensory predictions, the specific nature of the underlying mechanisms, particularly whether they necessarily involved top‐down modulation, remained an open question. Our finding provides clearer evidence for top‐down processing. It is important to clarify our use of the term “top‐down”. Although this term is often used in an anatomical sense to describe feedback from higher‐order regions (e.g., frontal cortex), a broader, functional definition characterizes “top‐down” influences as any modulation of sensory processing that is not driven by the immediate sensory input, but by internal states such as learned predictions. We adopt this functional definition here. Our findings fit this framework because the activation of the visual cortex by a predictive auditory cue, in the complete absence of a visual stimulus, is a neural response that cannot be explained by bottom‐up sensory information alone.

This cross‐modal influence, where a learned predictive signal from one modality (auditory) actively shapes processing in a primary sensory cortex of another modality (visual), is a hallmark of top‐down processing (for a review, see Emberson 2019). This suggests a hierarchical process where information about the learned audio‐visual association (likely stored in higher‐order association areas, though not directly measured) influences the visual system. The learned auditory cues’ impact on the visual cortex indicates the involvement of feedback neural connections (e.g., Emberson et al. 2015; Xiao and Emberson 2023). This demonstration of cross‐modal predictive modulation of a primary sensory cortex suggests that neonates are not only capable of sensory prediction but can also employ more complex neural mechanisms, such as top‐down processing, earlier in development than previously understood.

The finding that neonates were engaging in top‐down sensory prediction within 2 days after birth significantly challenges previous assumptions about the developmental timeline of such cognitive and neural capacities. Previous research across various domains posited that top‐down processing and prediction emerge and evolve over time (Amso and Sherif 2015; Christiansen and Chater 2016). In contrast, this new finding suggests that certain aspects of top‐down processing and prediction, particularly those influencing perceptual cortices, are present very early in development. However, this revelation does not address how this ability might develop further or vary in its application to different experiences or stimuli. For example, in examining top‐down sensory prediction of faces, Xiao, Emberson and colleagues found both developmental differences (Xiao and Emberson 2019) and differential abilities for applying top‐down sensory prediction for familiar versus unfamiliar stimuli (own vs. other race faces, Xiao et al. 2024). Thus, future work is needed to examine both how these early capacities support future development as well as how these abilities changes with development.

The finding of top‐down sensory prediction evokes the question of whether this kind of cognitive and neural capacity is innate or foundational to perception, cognition, and development. Although the current study does not definitively answer whether this ability is innate, it clearly indicates that the capacity for prediction and top‐down processing is accessible to full‐term infants from the earliest stages of postnatal development. It is noteworthy that infants born very and extremely prematurely do not show signatures of top‐down sensory prediction at 6 months corrected age (Emberson et al. 2017) suggesting that this capacity is not universally available and appears to be disrupted by premature birth.

The answer to the question of whether this ability is innate or foundational also relates to the question of the state of the visual system at birth. Neonates experience a dramatic shift in the sensory input for all senses with birth and perhaps vision is the greatest shift. Some studies, such as an MEG study by Eswaran et al. (2004), have found evoked visual responses in fetuses as early as 28 weeks gestational age, becoming more consistent by 32 and 36 weeks. Other research, like Reid et al. (2017), has indicated potential face‐specific behavioral responses in fetuses. Furthermore, an fMRI study showed hemodynamic responses to visual stimuli in fetuses (Fulford et al. 2003, see review of fetal functional imaging from Anderson and Thomason 2013). Thus, there is emerging evidence that fetuses are capable of responding to visual stimuli before birth. However, it remains unclear how much visual perception they are engaging in and whether there is learning taking place that would support the emergence of top‐down sensory prediction for visual stimuli based on these experiences. Future research focusing on the fetal visual environment could be instrumental in determining whether similar learning and prediction contexts are available to fetuses. Regardless, while it is not certain if the top‐down capabilities demonstrated by neonates here predate any experience, the current findings strongly suggest that minimal or even no prior experience is required for the neonatal brain to engage in top‐down sensory prediction of visual stimuli. This insight contributes significantly to our understanding of the early development of perceptual and cognitive processes.

A particularly noteworthy finding was the absent occipital response observed in the sA+V+ condition in Experiment 1, where a predicted visual stimulus was presented for six events, especially when contrasted with the significant visual cortical activation in the A+V− condition of Experiment 1, where an identical number of auditory cues predicted an absent visual stimulus. This pattern invites consideration of at least two plausible, and not necessarily mutually exclusive, interpretations. One perspective focuses on the characteristics of the sensory stimulation and maturational state of neonatal sensory processing. The relatively brief duration of visual stimulus presentation in sA+V+ might have been insufficient to elicit a robust, detectable hemodynamic response, particularly when compared to fNIRS paradigms in neonates that often employ longer stimulus exposures (e.g., 30 seconds; Benavides‐Varela et al. 2012; Gervain et al. 2008; Nallet et al. 2023). In this light, the significantly stronger visual response to the brief presentation of predictive auditory cues in the A+V− condition (Experiment 1), which effectively signaled a violated expectation in the absence of external visual input, is striking. This apparent discrepancy could suggest that, at this early developmental stage, internally generated signals related to expectation violation might evoke more pronounced cortical responses than brief, externally presented (even if predicted) visual stimuli. This aligns with the notion that top‐down modulatory influences, supported by anatomically extensive feedback pathways (Gilbert and Li 2013), might play a particularly salient role in early life before bottom‐up pathways are fully developed.

An alternative, yet potentially complementary, framework is offered by predictive coding theory (Friston 2005; Rao and Ballard 1999). Within this model, neural activity in sensory cortices primarily reflects the mismatch between top‐down predictions and bottom‐up sensory input (prediction error). Although it may seem counterintuitive for the unexpected *absence* of a stimulus to evoke a significantly stronger response than its *presence* (as seen in the A+V− vs. sA+V+ comparison), this pattern is a core feature of a predictive brain. The robust response in the A+V− condition would represent a strong prediction error signal when the expected visual stimulus was omitted.

In contrast, the attenuated response in the sA+V+ condition, where the auditory cue correctly predicted the visual event, can be interpreted as predictive suppression. Rather than simply reflecting a met expectation, top‐down predictions may actively dampen the response to an incoming sensory stimulus. This form of signal attenuation in response to predictable events has been observed in infants. For example, a recent study found that cortical activity in sensory regions was attenuated when 6‐month‐old infants viewed predictable temporal sequences (e.g., Baek et al. 2022). Therefore, the minimal activity in the sA+V+ condition is not necessarily a failure to respond, but rather evidence of a successful prediction leading to a suppressed bottom‐up signal. This interpretation explains the seemingly paradoxical finding that an omission can cause a larger neural response than a physically present, but fully predicted, stimulus. The more substantial response observed in the A+V+ condition (18 audiovisual events), despite also involving met predictions, might then be attributed to factors such as the summation of residual, unattenuated signals over a larger number of events, or the possibility that nascent top‐down predictive mechanisms in neonates do not yet achieve complete suppression of strong, repeated bottom‐up inputs.

Disentangling these interpretations definitively will require further research. We acknowledge that our finding presents a surprising contrast between responses to prediction violation and fulfillment. Future studies could systematically vary the duration and predictability of stimuli to explore the interplay between stimulus‐driven responses and predictive processing in the neonatal brain. Nevertheless, both perspectives underscore the sophisticated nature of neonatal cortical processing, suggesting that even at birth, the brain is not merely a passive recipient of sensory information but actively engages in predictive and modulatory operations. The ongoing maturation of these systems, likely involving processes like synaptic pruning to refine feedback circuits (Huttenlocher and Dabholkar 1997), is crucial for the development of mature perception (e.g., Xiao et al. 2024).

Our findings demonstrate that learned auditory‐visual associations can induce top‐down modulation of the neonatal visual cortex. However, important questions remain regarding the precision and scope of these early mechanisms. Specifically, while our study established that the visual response during omission was contingent on prior learning experience, future research could further delineate the stimulus specificity of this learned top‐down effect. For instance, does visual cortex activation occur exclusively in response to the exact auditory cue predictively paired, or might the neonatal brain generalize this learned top‐down modulation to similar auditory stimuli once a cross‐modal predictive link has been established? Insights from adult neuroimaging studies suggest strong stimulus‐specific predictive modulation within visual cortex: responses in primary visual areas (e.g., V1) are selectively suppressed for predictably cued stimuli compared to unpredictive but equally frequent stimuli (e.g., Alink et al. 2010; Richter et al. 2018). Extending this logic to neonates, alternative experimental designs could directly compare occipital responses to auditory cues varying systematically in predictive validity. For example, comparing visual cortical modulation during omission trials for consistently predictive auditory cues versus explicitly non‐predictive or weakly predictive cues could clarify whether neonatal visual cortex similarly exhibits such specificity. Such comparisons would reveal whether neonatal top‐down modulation is a finely tuned, graded phenomenon sensitive to the statistical strength of predictive cues or represents a more categorical process activated by the formation of any learned association. Elucidating these aspects of stimulus specificity and predictive sensitivity in neonatal top‐down modulation will advance our understanding of the computational precision and generalization capabilities inherent in early predictive processing.

Another interesting area of future investigation is to pinpoint the neural source of the observed top‐down processes. Although our focus on the occipital cortex allowed direct measurement of these effects within the visual system, limitations in the spatial resolution and co‐registration capabilities of our fNIRS setup, as previously discussed, prevented precise localization beyond occipital channels. Moreover, the origin of top‐down signals could involve a widely distributed network of brain regions. Although prior research suggests a potential role for the frontal cortex in top‐down processing in infants and adults (e.g., Bar et al. 2006; Jaffe‐Dax et al. 2020), other higher‐order areas, such as association cortices involved in audiovisual integration, could also contribute as well as the basal ganglia or hippocampus (e.g., Kok et al. 2020; Turk‐Browne et al. 2009). Therefore, while we provided evidence of top‐down modulation in neonates, further research with more extensive sensor coverage and/or alternative neuroimaging techniques is needed to fully elucidate the neural substrates underlying these processes.

In sum, the current study presents the first evidence that neonates, born at full‐term, are capable of top‐down sensory prediction within 2 days of birth. Specifically, after learning an audiovisual association, the sound followed by an unexpected omission of the visual stimulus produces a large response in the occipital lobe revealing an effect of prediction in an early perceptual region. This finding established that top‐down processes (i.e., using feedback connections to modulate regions earlier in the cortical hierarchy) and prediction are influencing activity in the visual system starting early in life. This discovery aligns with and contributes to recent theoretical advancements in the field of perceptual development (e.g., Emberson 2019). It suggests that the capacity for top‐down modulation may be a foundational aspect of perception and development, present and functioning from very early in life.

## Author Contributions

**Naiqi G. Xiao**: conceptualization, methodology, software, data curation, supervision, formal analysis, validation, writing – original draft, writing – review and editing, visualization, project administration, investigation. **Claire E. Robertson**: investigation, project administration. **Lauren L. Emberson**: conceptualization, methodology, funding acquisition, writing – original draft, writing – review and editing, resources, project administration, supervision, investigation, formal analysis.

## Funding

This research was supported by grants from the National Institutes of Health (R00 4R00HD076166‐02), McDonnell Foundation (220020505), and the Natural Sciences and Engineering Research Council of Canada (NSERC) Discovery Grants (RGPIN‐2020‐07129).

## Ethics Statement

This study was approved by the Institutional review board of Princeton University and Penn Medicine: University of Pennsylvania Health System.

## Conflicts of Interest

All authors declare that they have no conflicts of interest.

## Permission to reproduce material from other sources

There is no material acquired from other sources.

## Data Availability

Anonymized fNIRS data and corresponding analysis scripts can be found on the OSF website (https://osf.io/zvgue/). These data and scripts will be accessible for anyone.
